# Efficacy and safety of *Abelmoschus moschatus* capsules combined with *tripterygium* glycoside tablets on diabetic nephropathy: A systematic review and meta-analysis

**DOI:** 10.3389/fphar.2022.936678

**Published:** 2022-09-12

**Authors:** Peipei Zhou, Zhenning Hao, Weilong Xu, Xiqiao Zhou, Jiangyi Yu

**Affiliations:** ^1^ Department of Endocrinology, Jiangsu Province Hospital of Chinese Medicine, Affiliated Hospital of Nanjing University of Chinese Medicine, Nanjing, China; ^2^ The First Clinical Medical College, Nanjing University of Chinese Medicine, Nanjing, China

**Keywords:** diabetic nephropathy, *Abelmoschus moschatus*, *Tripterygium* glycosides, systematic review, meta-analysis

## Abstract

**Background:** Diabetic nephropathy (DN) is one of the most serious chronic micro-vascular complications of diabetes and the leading cause of end-stage kidney disease (ESRD) worldwide, with reduced expectancy and quality of life and colossal financial and social burden worldwide. In spite of emerging treatments on DN, effective therapy on delaying the progression of DN is still lacking. In clinical practice, there are many studies focusing on *Abelmoschus moschatus* (AM) capsules together with *Tripterygium* glycoside (TG) tablets in the treatment of DN, and excellent results have been obtained.

**Objective:** The study aimed to evaluate the efficacy and safety of AM combined with TG in the treatment of DN.

**Methods:** Databases including PubMed, Web of Science, Cochrane Library, Embase, CNKI, WF, and VIP were searched from their inception to 1 March 2022. The “risk of bias” evaluation tool produced by the Cochrane Collaboration Handbook was used for evaluating the quality of the included studies. Revman 5.3 software was used for meta-analysis.

**Results:** Here, 11 studies with a total of 1,072 participants were included for this meta-analysis. Our results showed that AM combined with TG plus basic treatment could lower levels of 24 h-UP [MD = -0.18; 95% CI: (-0.21, -0.14); *p* < 0.00001], Scr [MD = -15.29; 95% CI: (-28.69, -1.88); *p* = 0.03], and BUN [MD = -1.18; 95% CI: (-1.69, -0.68); *p* < 0.00001]. Meanwhile, the adverse reaction rate increased in the combination group [RR = 1.88; 95% CI (1.26, 2.82); *p* = 0.002].

**Conclusion:** Current evidence suggests that AM combined with TG may be more effective in the treatment of DN, which will be highly beneficial to further theoretical discussion and practical clinical applications. However, the safety cannot be ignored because of nearly increasing 2-fold adverse events, although they can be mitigated through systematic treatment. Meanwhile, due to low quality of the included studies, great possibility of publication bias, and large heterogeneity among different studies, the results of our review should be evaluated with more prudence and high-quality RCTs are warranted to confirm this in the future.

**Systematic review Registration:** www.crd.york.ac.uk, identifier CRD42022344359.

## Introduction

Diabetic nephropathy (DN) or diabetic kidney disease (DKD), recognized as one of the most prevalent microvascular complications of diabetes mellitus (DM) and a dominant cause of the end-stage renal disease (ESRD), has brought a great threat to health along with severe financial and social burden worldwide (Bikbov et al., 2020; Johansen et al., 2021). According to epidemiology surveys, the morbidity of DN accounted for 30–50% of ESRD across the world (Ruiz-Ortega et al., 2020). Meanwhile, patients with DM approximately increased to 693 million by 2045 (Cho et al., 2018), which indicated more and more people developed into DN as time goes on. DN is characterized by decreasing the renal function with the leakage of proteins in the urine (Persson and Rossing, 2011). The pathogenesis still remains questionable although hyperglycemia, inflammation, oxidative stress, and endoplasmic reticulum stress are universally recognized (Samsu, 2021). Current clinical therapeutic methods mainly focused on the optimized control of hyperglycemia and hypertension (American Diabetes Association, 2019), and the latest trials demonstrated sodium-glucose co-transporter 2 inhibitors (SGLT2i) had robust and consistent renal protective effects (Perkovic et al., 2019). However, these were insufficient to cease or reverse the unrelenting progression of DN due to metabolic memory, defined as remembering the prior hyperglycemic environment even in the states of normal glucose afterward (Kumar et al., 2016). Because of complicated and unclear mechanisms, it is indispensable to seek potential treatment approaches to prevent or delay the course of DN.

Research on Chinese herbal medicine (CHM) and its bioactive ingredients has produced more potential possibilities and might be useful for the identification and validation of new biomarkers and drug targets for early detection and treatment of DN (Tang et al., 2021). They exhibit a pleiotropic action profile through multiple targets and multiple pathways (Meresman et al., 2021), by means of which the fundamental process in the pathogenesis of DN may be simultaneously affected, and higher efficacy and lower side effects will reach. According to the theory of traditional Chinese medicine (TCM), DN belongs to the category of disease with the name “Shenxiao”. The pathogenesis of DN in TCM is the spleen and kidney deficiency along with excessive dampness and heat; the latter one round into the conglutination of blood stasis afterward. Correspondingly, the main principle of treatment is heat-damp clearance, channel activation, and stasis dissolution. In China, *Abelmoschus moschatus* (AM) capsules and *Tripterygium* glycoside (TG) tablets are adapted to kidney disease; the former is of damp-heat syndrome (Yu et al., 2022), whereas the latter is of damp-heat syndrome along with blood stasis and channel obstruction (Han and Jiang, 2020).

AM, with the Chinese name of Huangkui capsule, is an extract from *Abelmoschus manihot* (L.) Medik. with total flavones as primary active ingredients (Ge et al., 2016; Liu et al., 2021). A mass of studies demonstrate that AM has anti-inflammation, anti-fibrosis, and anti-oxidative stress effects through varieties of pathways to suppress abnormal renal cell proliferation, alleviate renal tubular epithelial-mesenchymal transition and endoplasmic reticulum stress, and improve metabolic disorders, which inhibits the progression of DN at last (Ge et al., 2016; Wu et al., 2018; Han et al., 2019; Gu et al., 2021). TG, *Tripterygium wilfordii* Hook. f., Lei Gong Teng tablets as a Chinese name, also has the characteristics of anti-inflammatory functions and excellent abilities of anti-oxidation in preventing progression of DN (Wu et al., 2017; Wu et al., 2020; Wang et al., 2021a). Moreover, both of them have been widely potentially exploited *in vivo* and *vitro*, as well as in high-quality clinical trials, which achieve great advantages in the treatment of DN. Of note, a quantitative evidence synthesis showed that adverse events induced by TG were systemic and organ-specific associated with the combined intervention, drug dose, and course of medication (Ru et al., 2019). According to the drug instructions of TG (National Medicine Standard Z43020138; Hunan Xieli Pharmaceutical Co. Ltd.), in addition to strong treatment functions, it brings about lots of physiological adverse effects, such as digestion and blood, cardiovascular, and neural dysfunctions. Similarly, AM (National Medicine Standard Z19990040; Jiangsu Suzhong Pharmaceutical Group Co. Ltd.) has adverse effects on the digestion system and skin based on medication instructions. In recent years, a combination of AM with TG in treating DN has been increasingly elevated; thus, it is necessary to evaluate their efficacy and safety, which is the emphasis of this systematic review and meta-analysis.

## Materials and methods

This meta-analysis was conducted in agreement with the guidelines of the Preferred Reporting Items for Systematic Reviews and Meta-Analysis (PRISMA) (Moher et al., 2009). The protocol has been registered on PROSPERO with the registration number CRD42022344359.

### Database and search strategies

We searched for eligible trials in electronic databases, including PubMed, Web of Science, Cochrane Library, Excerpta Medica Database (Embase), China National Knowledge Infrastructure Database (CNKI), Wanfang Database (WF), and China Science and Technology Journal Database (VIP), from the earliest publication date available to 1 March 2022 with the restricted language of Chinese and English. In parallel, the reference lists of published systematic reviews and included trials would also be retrieved to obtain additional eligible clinical trials. The predefined search terms included “*Abelmoschus moschatus*”, and “*Tripterygium* glycosides” and “diabetic nephropathy” were searched in combination.

### Inclusion criteria

Studies were selected if they were eligible for criteria based on PICOS as follows: 1) participants: adults diagnosed with DN in accordance with the Kidney of Disease Outcomes Quality Initiative of National Kidney Foundation in 2007 (KDOQI Clinical Practice Guidelines and, 2007) or the Expert consensus on prevention and treatment of diabetic kidney disease (2014 version) in China (Expert consensus, 2014), especially conforming to any of the following conditions: massive proteinuria, diabetic retinopathy accompanied by CKD of any stage, and microalbuminuria that occurs in type 1 diabetes, where the course of diabetes is lasting for over 10 years. 2) Intervention: AM combined with TG orally and basic treatment (BT) applied. 3) Comparison: AM with BT. BT includes the adjustment of blood glucose, blood pressure, blood lipid, anti-infection, preservation of body fluids, electrolyte, and acid–base homeostasis. 4) Outcomes: primary outcome measures comprising 24 h urinary protein quantitation (24 h-UP) and urinary albumin excretion rates (UAERs), regardless of accompanying serum creatinine (Scr) or blood urea nitrogen (BUN) or the clinical improvement rate or adverse effective rate. 5) Study design: randomized clinical trials (RCTs) regardless of blinding, bias, or protocols.

### Exclusion criteria

Studies were excluded if they were not eligible for conditions as follows: 1) patients who received hemodialysis or peritoneal dialysis in stage V. 2) Other TCM characteristic therapies including acupuncture, TCM enema, and physical therapy in the intervention group. 3) No primary outcomes described. 4) Theoretical explorations, case reports, systematic review, observational research without a control group, and animal or cell experiments. 5) No RCTs. 6) Duplicated publications

### Data extraction

Relevant studies were carefully screened by two independent investigators on titles and abstracts after removing duplicates. Full texts were also obtained by two investigators. When disagreements occurred between two investigators, they would be resolved through consultation with a third investigator. The data were extracted independently and double-checked from the included studies, for instance, first author, year of publication, sample size, gender, average age, course of the disease, methodological details, treatment duration, and outcomes.

### Quality evaluation

Two investigators accessed the quality of the included studies separately according to the Cochrane Collaboration Handbook for Systematic Reviews of Intervention. It was sorted into three levels of bias: “low risk,” “unclear risk,” “high risk”. The following six domains were evaluated: 1) selection bias included random sequence and allocation concealment; 2) performance bias referred to blinding of participants and personnel; 3) detection bias equaled to blinding of the outcome assessment; 4) attrition bias was the incomplete outcome data; 5) reporting bias referred to selective reporting; 6) bias of other resources. Divergences were resolved by discussing and reaching a consensus with a third investigator if necessary.

### Statistical analysis

Review Manager 5.3 was used for graphing and accessing the results from the aforementioned evaluations. The pooled risk ratio (RR) with a 95% confidence interval (CI) was used for assessing discontinuous variables; otherwise, the pooled mean difference (MD) with 95% CI served as continuous variables. The *p*-value was used for calculating outcomes, and when less than 0.05, it was recognized as statistically significant. The I^2^ statistic was used to evaluate heterogeneity among the included studies. I^2^>50% was indicative of high heterogeneity, and the random-effect model was applied; if not, the fixed model was employed. Meanwhile, sensitivity analysis was also performed by deleting included studies one by one to testify to the robustness. In order to analyze the possible sources of heterogeneity, subgroup analyses were conducted based on treatment duration, average age, and baseline of Scr. Funnel plots were employed for examining potential publication bias when more than 10 studies were brought into the analysis.

### Search results

**FIGURE 1:**
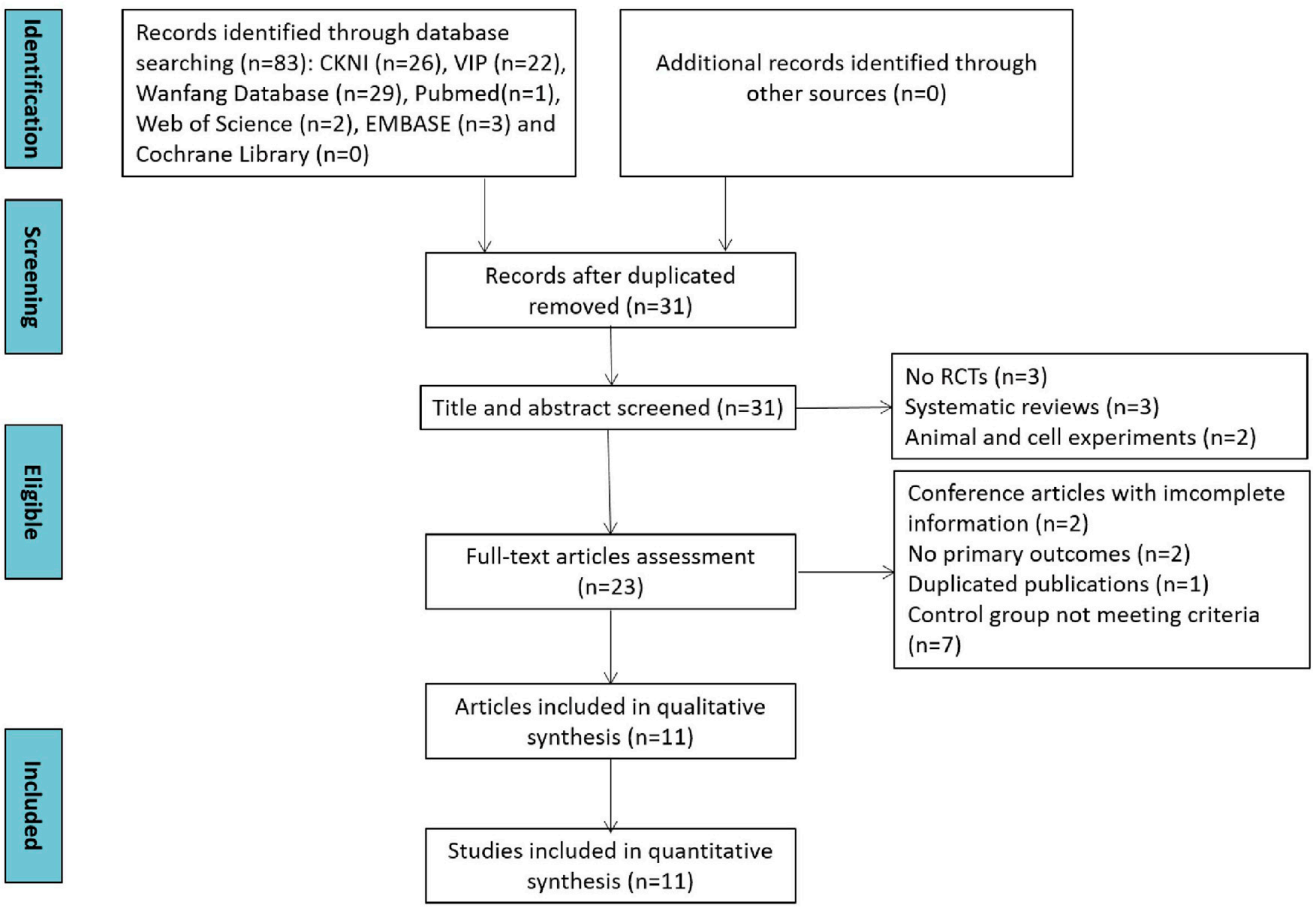
Flow diagram of literature screening.

In total, 83 studies were identified through database searching. First, 31 studies were screened out by eliminating duplication. Second, eight studies were removed due to no RCT trials and systematic reviews after screening title and abstracts. Then, 12 studies were swept away because of obtaining incomplete information, no primary outcomes, duplicated articles, and not meeting the criteria of the control group. Ultimately, 11 studies were included in the qualitative and quantitative synthesis. The study selection process is depicted in Figure 1.

### Study characteristics

Eleven studies with a total of 1,072 participants (538 from the intervention group and 534 from the control group) were included for meta-analysis after careful screening and evaluation. All included studies were single-centered published from 2010 to 2021 and conducted in China. The sample size ranged from 64 to 180, and the average course ranged from 3.21 to 10.2 years in every recorded study. The mean age was 55.39 years old in the intervention group and 55.74 years old in the control group with 549 males and 433 females, except for one study (Shen et al., 2011) with unclear data. Meanwhile, the intervention duration lasted from 8 to 12 weeks. More detailed characteristics are presented in Table 1.

**TABLE 1:** Basic characteristics of the included studies.

Study	Basic characteristics (I/C)			Gender (M/F	Intervention measures	Duration (w)	Mean baseline levels (I/C)		Outcome
	Sample size	Mean age (y)	Course (y)				24 h-UP	Scr
Du and Gao (2011)	34/30	48.85/49.36	NM	33/31	I: AM (2.5 g, tid); TG (10 mg, tid); LS; LP; LL C: AM (2.5 g, tid); LS; LP; LL	8	1.52/1.45	137.3/128.1	O1,3,4,5,6
Ge et al. (2020)	52/52	56.86/57.31	NM	59/45	I: AM (2.5 g, tid); TG (20 mg, tid); LS; LP; DS C: AM (2.5 g, tid); LS; LP; DS	12	0.64/0.64	195.07/197.25	O1,3,4,5,6
Li (2012)	64/64	45.3/46.2	3.7/3.4	69/59	I: AM (2.5 g, tid); TG (20 mg, tid); LS; LP C: AM (2.5 g, tid); LS; LP	12	NM	NM	O2,5,6
Shen et al. (2011)	45/45	56	8.1	unclear	I: AM (2.5 g, tid); TG (1 mg/kg/d, tid); LS; LP C: AM (2.5 g, tid); LS; LP	12	3.0/3.0	91.7/90.2	O1,3,6
Shi (2018)	41/41	51.61/52.31	4.82/5.12	52/30	I: AM (2.5 g, tid); TG (20 mg, tid); LS C: AM (2.5 g, tid); LS	12	2.95/2.86	83.21/81.46	O1,3,4,6
Su (2021)	40/40	65.26/64.35	NM	45/35	I: AM (2.15 g, tid); TG (20 mg, tid); LS; LP C: AM (2.15 g, tid); LS; LP	12	NM	NM	O1,3,4
Wang et al. (2021b)	34/34	61.3/61.2	8.9/8.5	41/27	I: AM (2.5 g, tid); TG (20 mg, tid); LS; LP C: AM (2.5 g, tid); LS; LP	12	NM	NM	O1,3,4,6
Xu et al. (2016)	63/63	58.6/59.4	8.6/9.1	82/44	I: AM (2.5 g, tid); TG (40 mg, tid for 4 weeks and then reduce to 20 mg, tid); LS; LP; LL C: AM (2.5 g, tid); LS; LP; LL	8	1.79/1.88	127.30/128.12	O1,3,4,5,6
Yang (2021)	41/41	53.24/54.06	3.24/3.17	45/37	I: AM (2.5 g, tid); TG (20 mg, tid); LS C: AM (2.5 g, tid); LS	12	2.94/2.97	170.57/169.35	O1,3,4,5
Zhang et al. (2010)	34/34	58.6/59.4	10.6/9.8	37/31	I: AM (5 pills, tid); TG (20 mg, tid); LS; LL; DS C: AM (5 pills, tid); LS; LL; DS	12	4.75/4.73	133.25/133.16	O1,3,4,5,6
Zhu et al. (2018)	90/90	56.5/56.1	8.4/8.1	86/94	I: AM (5 pills, tid); TG (1 mg/kg/d, tid); LS; LP C: AM (5 pills, tid); LS; LP	12	2.9/2.9	91.6/90.1	O1,3,6

I, intervention group; C, control group; AM, *Abelmoschus moschatus*; TG, *Tripterygium* glycoside tablets; LS, lower blood sugar; LP, lower blood pressure; LL, lipid-lowering; DS, diuresis; 24 h-UP: 24 h urinary protein quantitation; Scr, serum creatinine; NM, not mentioned; y, year; w, week; tid, three times a day; NM, not mentioned; O1, 24 h-UP; O2, UAER; O3, scr; O4, blood urea nitrogen; O5, clinical improvement rate; O6, adverse effective rate.

### Quality assessment of included studies

**FIGURE 2:**
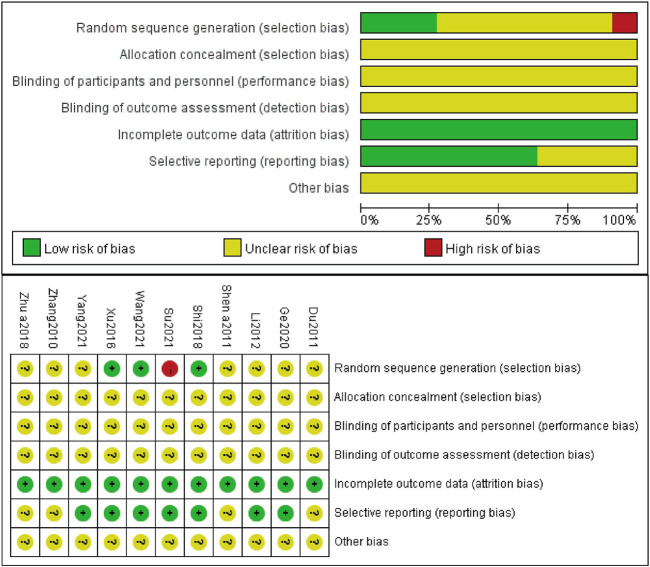
Risk of bias graph and risk of bias summary.

Three studies (Xu et al., 2016; Shi, 2018; Wang et al., 2021b) made random sequence generation by using a random number table. However, one study (Su, 2021) utilized a random sampling method, which was inappropriate and considered a high risk. The rest of included studies did not describe the process of generating random sequences and were recognized as unclear risks. Seven studies (Li, 2012; Xu et al., 2016; Shi, 2018; Ge et al., 2020; Wang et al., 2021b; Su, 2021; Yang, 2021) previously prepared informed consent of patients along with their family members and approval by the Ethics Committee of the affiliated hospital, either or both of which was acquired, so bias of which was judged as low risk. None of the included studies implemented allocation concealment, blinding of participants, personnel and outcome assessment, and other bias. The results of the quality assessment of included studies are shown in Figure 2.

## Results

### 24 h-UP

**FIGURE 3:**
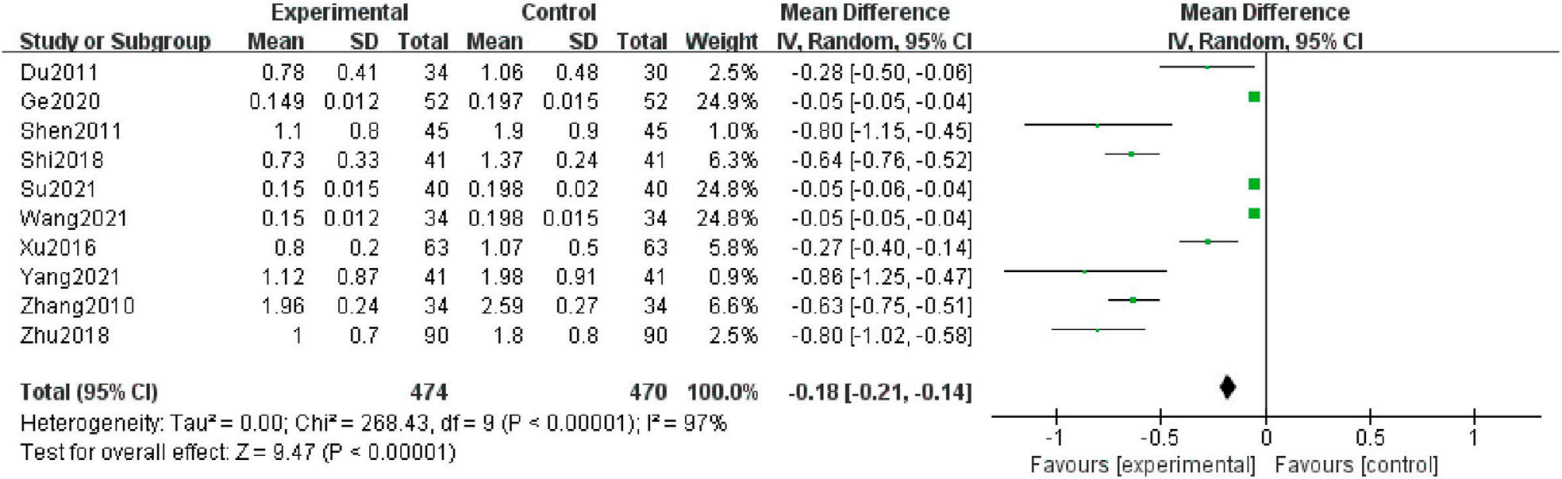
Forest plot of 24 h-UP.

Ten studies (Zhang et al., 2010; Du and Gao, 2011; Shen et al., 2011; Xu et al., 2016; Shi, 2018; Zhu et al., 2018; Ge et al., 2020; Wang et al., 2021b; Su, 2021; Yang, 2021), including 944 patients reported the 24 h-UP levels. Due to high heterogeneity (I^2^ = 97%), a random-effect structure was conducted, and the result showed that it was significantly decreased in the combination group compared with AM [MD = -0.18; 95% CI: (-0.21, -0.14); *p* < 0.00001] (Figure 3).

Meanwhile, subgroup analyses of 24 h-UP were conducted to explore the sources of high heterogeneity based on treatment duration, average age, and baseline of Scr (Table 2 and Supplementary Figures S1-S3). The heterogeneity of patients with treatment duration of 12 weeks, average age more than 60 years old, and the baseline of Scr less than 100 μmol/L and 100–133 μmol/L significantly decreased. However, high heterogeneity still existed in other groups during the same criteria for grouping, which indicated that average age, treatment duration, and baseline of Scr could be the sources of heterogeneity.

**TABLE 2:** Subgroup analyses of 24 h-UP based on treatment duration, average age, and baseline of Scr.

Criteria for grouping	Subgroup	n	MD (95%)	I^2^ (%)	Z	*p*
Treatment duration	8 weeks	8	-0.16 [-0.20, -0.13]	97	8.61	<0.00001
	12 weeks	2	-0.27 [-0.39, -0.16]	0	4.69	<0.00001
Average age	<50 years old	1	-0.28 [-0.50, -0.06]	–	2.49	0.01
	50–60 years old	7	-0.56 [-0.86, -0.26]	98	3.68	0.0002
	>60 years old	2	-0.05 [-0.05, -0.04]	0	18.97	<0.00001
Baseline of Scr	<100 μmol/L	3	-0.69 [-0.79, -0.59]	0	13.03	<0.00001
	100–133 μmol/L	2	-0.27 [-0.39, -0.16]	0	4.69	<0.00001
	>133 μmol/L	3	-0.49 [-0.99, 0.01]	98	1.93	0.05

### Scr

**FIGURE 4:**
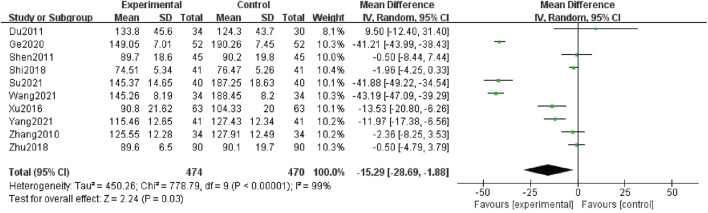
Forest plot of Scr.

Ten studies (Zhang et al., 2010; Du and Gao, 2011; Shen et al., 2011; Xu et al., 2016; Shi, 2018; Zhu et al., 2018; Ge et al., 2020; Wang et al., 2021b; Su, 2021; Yang, 2021), comprising 944 patients, talked of Scr as an outcome. Because of high heterogeneity (I^2^ = 99%), a random-effect structure was utilized, and the result showed that levels of Scr in the combination group were significantly decreased compared with AM [MD = -15.29; 95%CI: (-28.69, -1.88); *p* = 0.03] (Figure 4).

Furthermore, we looked for the sources of high heterogeneity on Scr *via* subgroup analyses based on treatment duration, average age and baseline of Scr (Table 3 and Supplementary Figures S4-S6). The heterogeneity of patients in the group of average age more than 60 years old and baseline of Scr less than 100 μmol/L obviously decreased. Interestingly, the patients between the combination group and control group presented no significant differences in the subgroups of treatment duration with 12 weeks [MD = -4.43; 95% CI: (-26.49, 17.64); *p* = 0.69], average age <50 years old [MD = 9.50; 95% CI: (-12.40, 31.40); *p* = 0.40] and 50–60 years old [MD = -10.35; 95% CI: (-25.28, 4.58); *p* = 0.17], baseline of Scr <100 μmol/L [MD = -1.57; 95% CI: (-3.53, 0.39); *p* = 0.12], 100–133 μmol/L [MD = -4.43; 95% CI: (-26.49, 17.64); *p* = 0.69] and >133 μmol/L [MD = -18.61; 95% CI: (-44.43, 7.21); *p* = 0.16].

**TABLE 3:** Subgroup analyses of Scr based on treatment duration, average age, and baseline of Scr.

Criteria for grouping	Subgroup	n	MD (95%)	I^2^ (%)	Z	*p*
Treatment duration	8 weeks	8	-17.97 [-33.16, -2.78]	99	2.32	0.02
	12 weeks	2	-4.43 [-26.49, 17.64]	74	0.39	0.69
Average age	<50 years old	1	9.50 [-12.40, 31.40]	–	0.85	0.40
	50–60 years old	7	-10.35 [-25.28, 4.58]	99	1.36	0.17
	>60 years old	2	-42.90 [-46.34, -39.46]	0	24.43	<0.00001
Baseline of Scr	<100 μmol/L	3	-1.57 [-3.53, 0.39]	0	1.57	0.12
	100–133 μmol/L	2	-4.43 [-26.49, 17.64]	74	0.39	0.69
	>133 μmol/L	3	-18.61 [-44.43, 7.21]	99	1.41	0.16

### BUN

**FIGURE 5:**
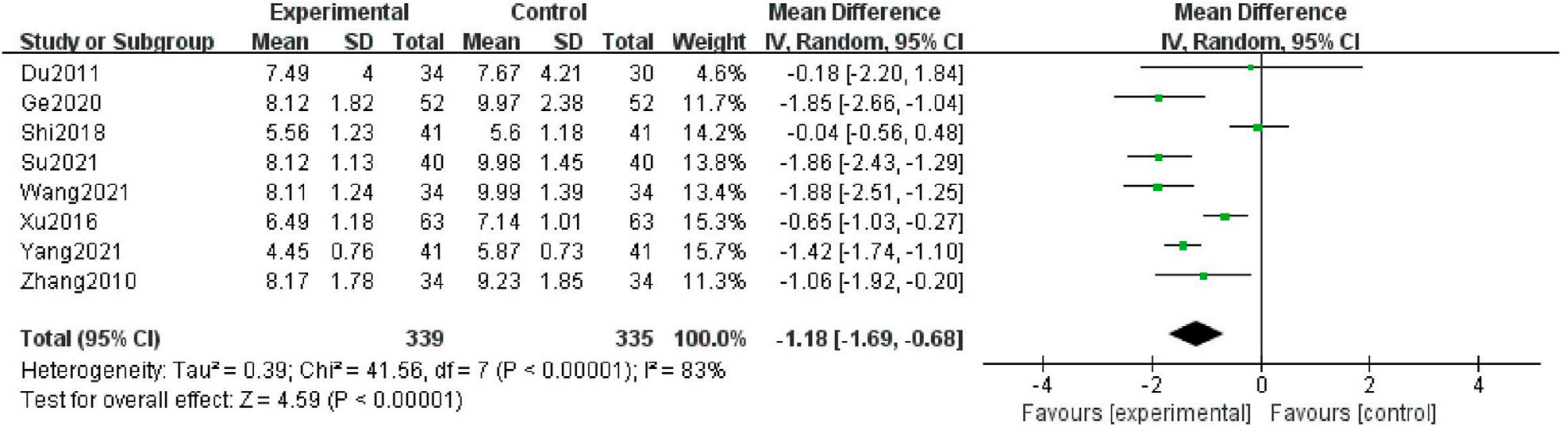
Forest plot of BUN.

Eight studies (Zhang et al., 2010; Du and Gao, 2011; Xu et al., 2016; Shi, 2018; Ge et al., 2020; Wang et al., 2021b; Su, 2021; Yang, 2021) contributed to this analysis, and 674 patients were included. The data were analyzed by a random-effect structure because the heterogeneity was high (I^2^ = 83%). The meta-analysis indicated that in comparison with the control group, the combination group could further decline in levels of BUN [MD = -1.18; 95% CI: (-1.69, -0.68); *p* < 0.00001] (Figure 5).

Moreover, we conducted subgroup analyses according to the following criteria for grouping: treatment duration, average age, and baseline of Scr (Table 4 and Supplementary Figures S7-S9). Heterogeneity in a treatment duration of 12 weeks, average age more than 60 years old, the baseline of Scr 100–133 μmol/L, and more than 133 μmol/L significantly reduced. However, combination treatment did not demonstrate significant differences compared with the control group in subgroup analyses of grouping in average age less than 50 years old [MD = -0.18; 95% CI: (-2.20, 1.84); *p* = 0.86] and baseline of Scr less than 100 μmol/L [MD = -0.04; 95% CI: (-0.56, 0.48); *p* = 0.88].

**TABLE 4:** Subgroup analyses of BUN based on treatment duration, average age, and baseline of Scr.

Criteria for grouping	Subgroup	n	MD (95%)	I^2^ (%)	Z	*p*
Treatment duration	8 weeks	6	™1.34 [™1.93, ™0.75]	84	4.46	<0.00001
	12 weeks	2	™0.63 [™1.01, ™0.26]	0	3.30	0.001
Average age	<50 years old	1	™0.18 [™2.20, 1.84]	–	0.17	0.86
	50–60 years old	4	™0.83 [™1.50, ™0.17]	79	2.46	0.01
	>60 years old	3	™1.63 [™1.95, ™1.31]	27	9.87	<0.00001
Baseline of Scr	<100 μmol/L	1	™0.04 [™0.56, 0.48]	–	0.15	0.88
	100–133 μmol/L	2	™0.63 [™1.01, ™0.26]	0	3.30	0.001
	>133 μmol/L	3	™1.43 [™1.72, ™1.15]	0	9.92	<0.00001

### Clinical improvement rate

Six studies (Zhang et al., 2010; Du and Gao, 2011; Li, 2012; Xu et al., 2016; Ge et al., 2020; Yang, 2021) described the clinical improvement rate, covering a total of 572 patients. Due to the low heterogeneity reported (I^2^ = 10%), a fixed-effect structure was used to analyze the data. The combination group could further improve the clinical improvement rate compared with the control group [RR = 1.25, 95% CI (1.15, 1.37), *p* < 0.00001] (Figure 7).

**FIGURE 6:**
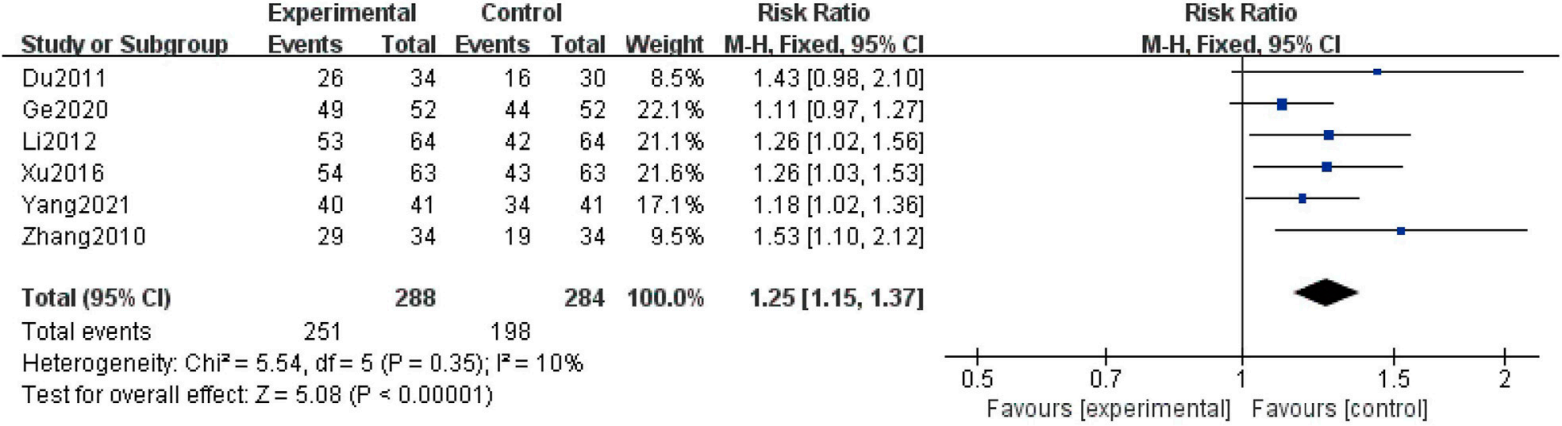
Forest plot of the clinical improvement rate.

**FIGURE 7:**
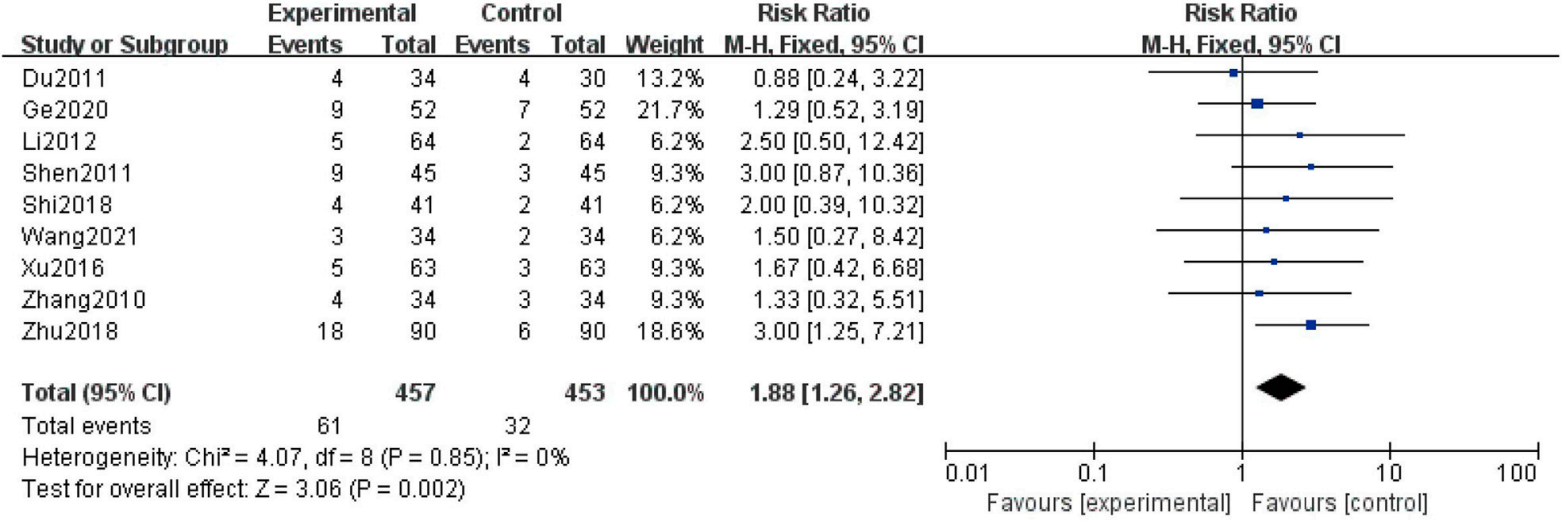
Forest plot of the adverse reaction rate.

### Safety assessment

Nine studies (Zhang et al., 2010; Du and Gao, 2011; Shen et al., 2011; Li, 2012; Xu et al., 2016; Shi, 2018; Zhu et al., 2018; Ge et al., 2020; Wang et al., 2021b) evaluated the safety, including 910 patients. The heterogeneity remained low (I^2^ = 0%); therefore, we conducted analysis by using a fixed-effect structure. The meta-analysis indicated that the combination group showed a higher adverse reaction rate in comparison with the control group [RR = 1.88; 95% CI: (1.26, 2.82); *p* = 0.002](Figure 7). The statistic of adverse reaction events is summarized in Table 5.

**TABLE 5:** Statistic of adverse reaction events.

Adverse reaction event	Intervention group (457 patients)	Control group (453 patients)
Gastrointestinal reactions	40 (8.75%)	31 (6.84%)
Abnormal liver enzymes	7 (1.53%)	1 (0.22%)
Leukopenia	14 (3.06%)	0
Total	61 (13.35%)	32 (7.06%)

### Sensitivity analysis

Sensitivity analysis was conducted by omitting each study in turn, which aimed to check the robustness of each outcome, including 24 h-UP, Scr, and BUN. The analysis indicated that the pooled MD and RR were stable, so we conducted a meta-analysis in all studies.

### Publication bias

**FIGURE 8:**
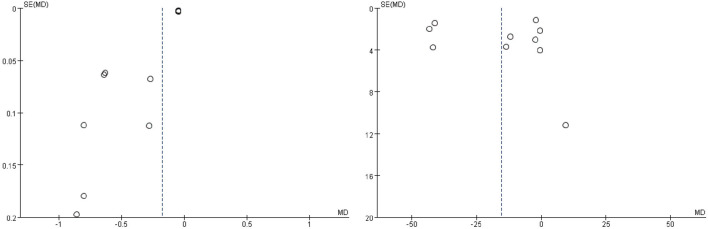
Funnel plots of 24 h-UP (left) and Scr (right).

No strictly symmetrical patterns were presented in the funnel plots of 24 h-UP and Scr (Figure 8). As to others, we did not evaluate the publication bias because of no more than 10 studies.

## Discussion

### Summary

TCM, especially AM and TG, are increasingly used in combination with the regulation of glucose, blood pressure, and electrolyte disturbance and broadly prescribed for DN patients in recent years. To evaluate whether the combination of AM and TG with BT improves the clinical efficacy and safety, 11 studies composed of 1,072 patients were included in this meta-analysis. To the best of our knowledge, AM combined with TG and BT in the treatment of DN is the first systematic review and meta-analysis that has evaluated clinical efficacy and safety in comparison with AM and BT.

### Effectiveness

24 h-UP is the quantity of urinary albumin excretion in 24 h. The incidence of progressing to ESRD in patients with moderately increased albuminuria is 9.3 times than that of those with normoalbuminuria (Berhane et al., 2011); meanwhile, baseline albuminuria and future cardiovascular events present a progressive relationship (Gerstein et al., 2001). Our results indicated that AM combined with TG in the intervention group could decrease the levels of 24 h-UP. Furthermore, subgroup analyses showed that sources of heterogeneity might come from average, treatment methods, and baseline of Scr. Therefore, the combination of AM and TG benefits a lot in decreasing the levels of 24 h-UP, although the included studies are not high. Nevertheless, more in-depth studies are also warranted in the future.

Scr and urinary albumin excretion are the earliest manifestations of DN (KDIGO 2020 Clinical Practice Guideline, 2020). Scr and BUN have commonly used indicators of injury renal function. Studies on the evaluation of TG or AM with BT in treating DN did not show significant advantages (Hu et al., 2019; Cheng et al., 2022). We found that the levels of Scr decreased in the combination group of AM and TG, and the average age and baseline of Scr could be the sources of high heterogeneity. Many subgroup analyses showed negative results in a treatment duration of 12 weeks, average age less than 50 years old, and baseline of Scr of all subgroups. All of the included studies in subgroups are not more than 3; thus, these conclusions are relatively unreliable and needed to be verified by further studies. Moreover, the subgroup analysis of average age between 50 and 60 and even less than 50 years old showed no obvious differences between the two groups. Supposing these results are reliable, patients less than 60 years old may not have a severe initial state of illness and would have better self-healing or bioavailability. In addition, the fact that no high-quality included studies and considerable high heterogeneity still existed in the results of other subgroups is not to be overlooked. However, further studies are needed to ascertain, and cautious altitudes should be taken to the results of this outcome.

However, controversial results existed in the combination group of AM or TG with BT in the evaluation of BUN compared with BT (Hu et al., 2019; Zhu et al., 2019; Wang et al., 2021c; Cheng et al., 2022). Our results turned out that compared with the control group, levels of BUN declined in the combination group, and consistent conclusions were obtained in subgroups of treatment duration, average age, and baseline of Scr. Negative results were observed in subgroups of the average age of less than 50 years old and baseline of Scr less than 100 μmol/L. But these two subgroup analyses contain only one study, respectively, and the validity of conclusions is warranted to confirm by further studies.

In conclusion, a combination of AM and TG with BT in the treatment of DN significantly increases the clinical improvement rate and decreases the levels of 24 h-UP and BUN, which is mostly in accordance with subgroup analyses based on treatment duration, average age, and baseline of Scr. As to Scr, although negative results emerged, the included studies in every subgroup are few, and more studies are needed to confirm. From the aforementioned analysis, all of these factors may be the sources of heterogeneity, and only the baseline of Scr is the one ascertained source of heterogeneity on BUN. However, the possibility of detection equipment and techniques in different hospitals and sample sizes, which result in high heterogeneity, cannot be ruled out. Regarding negative results between the combination and control group, we cannot exclude the possibility of patients less than 60 years old owning better self-healing and bioavailability, lighter states of an illness easier to recover, and insufficient sample size cannot be ignored as well. Anyway, the included studies are not high, so more cautious attitudes need to be devoted, and these results require further corroboration by clinical trials of multi-center, random, and blinding types in the future.

### Safety

In this meta-analysis, the combination group increased the adverse events in the treatment of DN, which needed special attention. The adverse events mainly focused on gastrointestinal reactions, abnormal liver enzymes, and leukopenia, although they returned to normal after corresponding symptomatic treatment without affecting the course of medication. Multi-center clinical trials and meta-analysis reported that AM in the treatment of primary glomerular disease, IgA nephropathy, and CKD all have no severe events alone or combined in contrast to renin-angiotensin system (RAS) inhibitors (Zhang et al., 2014; Li et al., 2020; Sun et al., 2022), which was in consistency with this article and suggested that the adverse events mainly came from TG. TG, with excellent therapeutic ability, in spite of the non-negligible issue of toxicity, deserves to be taken the risk of applying it to clinical treatment. As mentioned previously, TG-induced adverse events are systematic and organ-specific and affected by drug dosage, medication course, and combined intervention (Ru et al., 2019); thus, safe dosage and medication course for 3 months are more recommended. A group of 60 mg/d has shown no significant differences from a group of 30 mg/d; meanwhile, they were more effective in clinical efficacy after 6 months (Wang, 2018). No significant differences are detected between medication courses of 3 and 6 months in safety profiles (Jiang et al., 2015). Nevertheless, a recent meta-analysis has shown that a combinatorial treatment regimen including TG improves pathological indicators for DN progression and simultaneously causes a high risk of severe adverse events, and even medication course was limited to 3 months and inevitable life-threatening events occur (Li et al., 2021). Moreover, fatal events proportionally increased as the TG treatment goes on. As to this meta-analysis, medication courses among the included studies are no more than 3 months, and the dosage of TG in all included studies is not over 60 mg/d ultimately. Hence, it is difficult to evaluate whether drug dosage or medication course of 6 months increases the adverse events. However, the incidence of adverse events was nearly twice that of the control group with no TG, and adverse effects during medication should be kept an eye on as well even in situations of safe dosage and treatment duration.

Up to now, although there are many meta-analyses researching AM or TG on the treatment of DN, only one study focus on AM together with TG versus TG (Gao, 2015). Compared with it, there are some differences existing in this meta-analysis. This article does not emphasize TG as control but emphasizes AM as control. It also explores more objective clinical indexes regarding kidney function, such as Scr and BUN, apart from 24 h-UP. Moreover, this article has more included studies and participants whereas it contains only five studies. Hence, this meta-analysis is more creditable and integrated for further accessing the efficacy and safety of AM combined with TG in the treatment of DN.

### Limitations and strengths

Some inevitable limitations exist in this meta-analysis, which are needed to be taken into further consideration. First of all, the language of included studies is restricted to Chinese and English, which leads to selection bias. Second, since no high-quality enough studies are included, serious attention should be paid to interpreting the results. Third, there are still great limitations on 24 h-UP, Scr, and BUN as surrogate end points to judge the progression of DN. Last but not the least, there is publication bias in the evaluation of Scr and BUN, so the results should be explained with caution. Hence, more studies with a multi-center, canonical methodology of random, blindness, allocation concealment, and reporting negative results in reality are urgently warranted in real-world research.

Although there are limitations mentioned earlier, this meta-analysis and systematic review still provide valuable insights. The combination of AM and TG on the basis of BT in treating DN is one of the most revealing findings of this meta-analysis, which is more effective and challenging. This is of clinical importance and a promising choice for patients with DN when symptomatic treatments are identified to be less effective.

## Conclusion

Current evidence suggests that *Abelmoschus moschatus* capsules combined with *Tripterygium* glycoside tablets may be more effective in the treatment of DN, which will be highly beneficial to further theoretical discussion and practical clinical applications. As to safety, more adverse events in the combination group cannot be ignored because of nearly increasing 2-fold adverse events than those of the control group with no TG, although they can be solved through corresponding systematic treatment. However, due to the low quality of the included studies, the great possibility of publication bias, and large heterogeneity among different studies, the results of our review should be evaluated with more prudence, and high-quality clinical RCTs are warranted to confirm this in the future.

## Data Availability

The original contributions presented in the study are included in the article/Supplementary Material; further inquiries can be directed to the corresponding author.

## Supplementary material

Supplementary Figure S1 Subgroup analysis of 24h-UP based on treatment duration.

Supplementary Figure S2 Subgroup analysis of 24h-UP based on average age.

Supplementary Figure S3 Subgroup analysis of 24h-UP based on baseline of Scr.

Supplementary Figure S4 Subgroup analysis of Scr based on treatment duration.

Supplementary Figure S5 Subgroup analysis of Scr based on average age.

Supplementary Figure S6 Subgroup analysis of Scr based on baseline of Scr.

Supplementary Figure S7 Subgroup analysis of BUN based on treatment duration.

Supplementary Figure S8 Subgroup analysis of BUN based on average age.

Supplementary Figure S9 Subgroup analysis of BUN based on baseline of Scr.
