# Supplementary figures and images for: Efficacy and safety of Abelmoschus moschatus capsules combined with tripterygium glycoside tablets on diabetic nephropathy: A systematic review and meta-analysis
Source: Front Pharmacol. 2022 Sep 12;13:936678. doi: 10.3389/fphar.2022.936678 (PMC9511110; doi:10.3389/fphar.2022.936678)

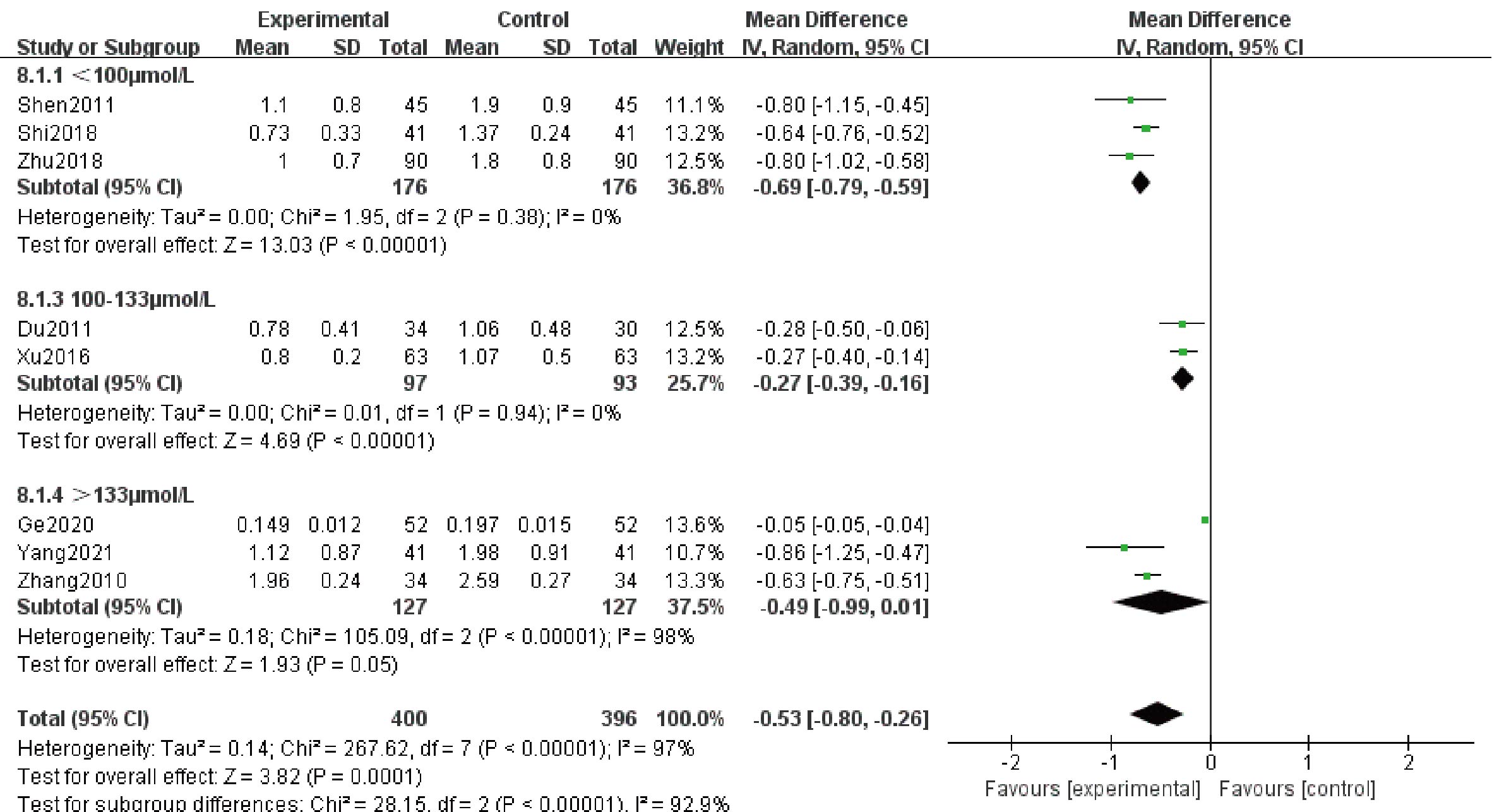

Supplement: Supplementary file 1 [file Image3.JPEG]

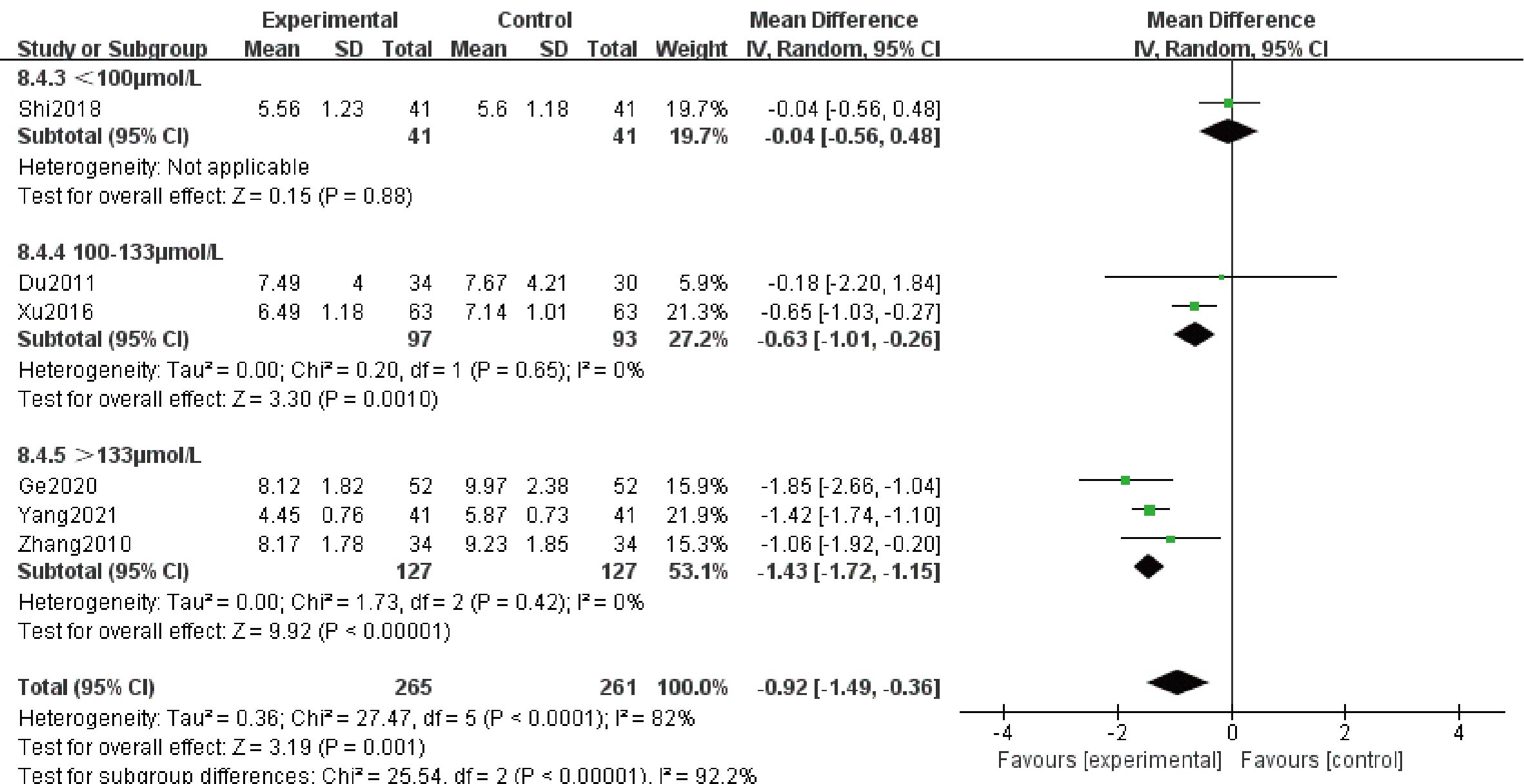

Supplement: Supplementary file 2 [file Image9.JPEG]

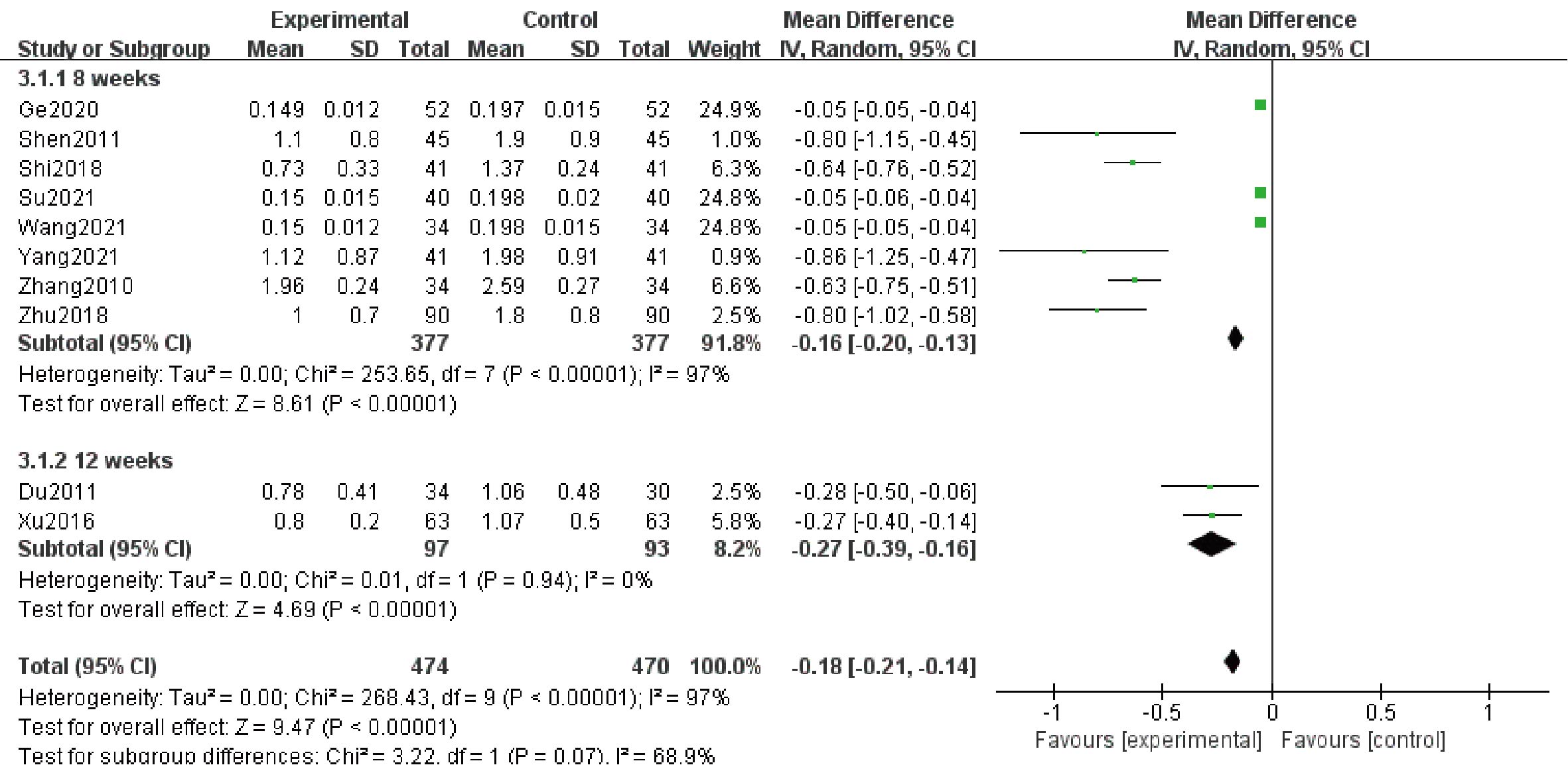

Supplement: Supplementary file 3 [file Image1.JPEG]

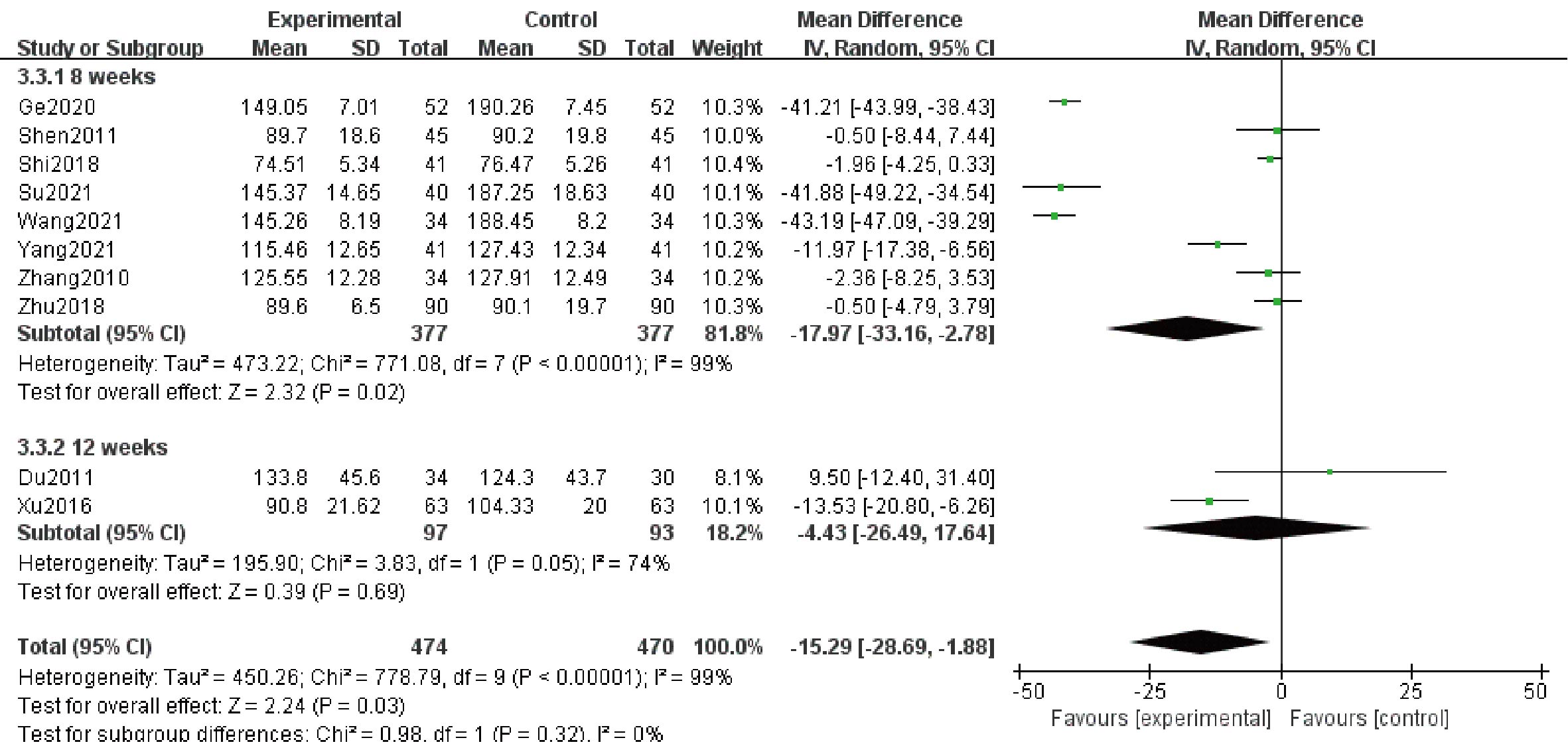

Supplement: Supplementary file 4 [file Image4.JPEG]

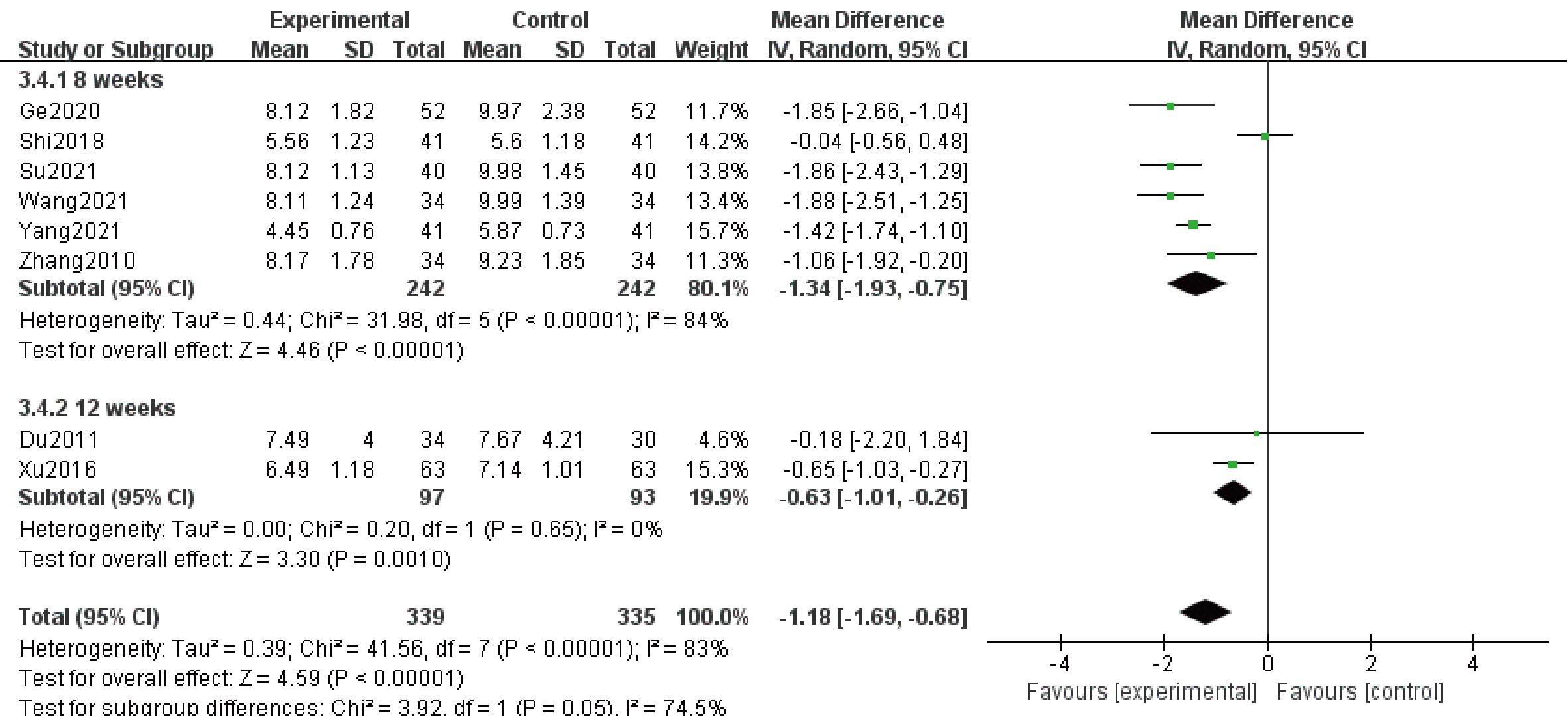

Supplement: Supplementary file 5 [file Image7.JPEG]

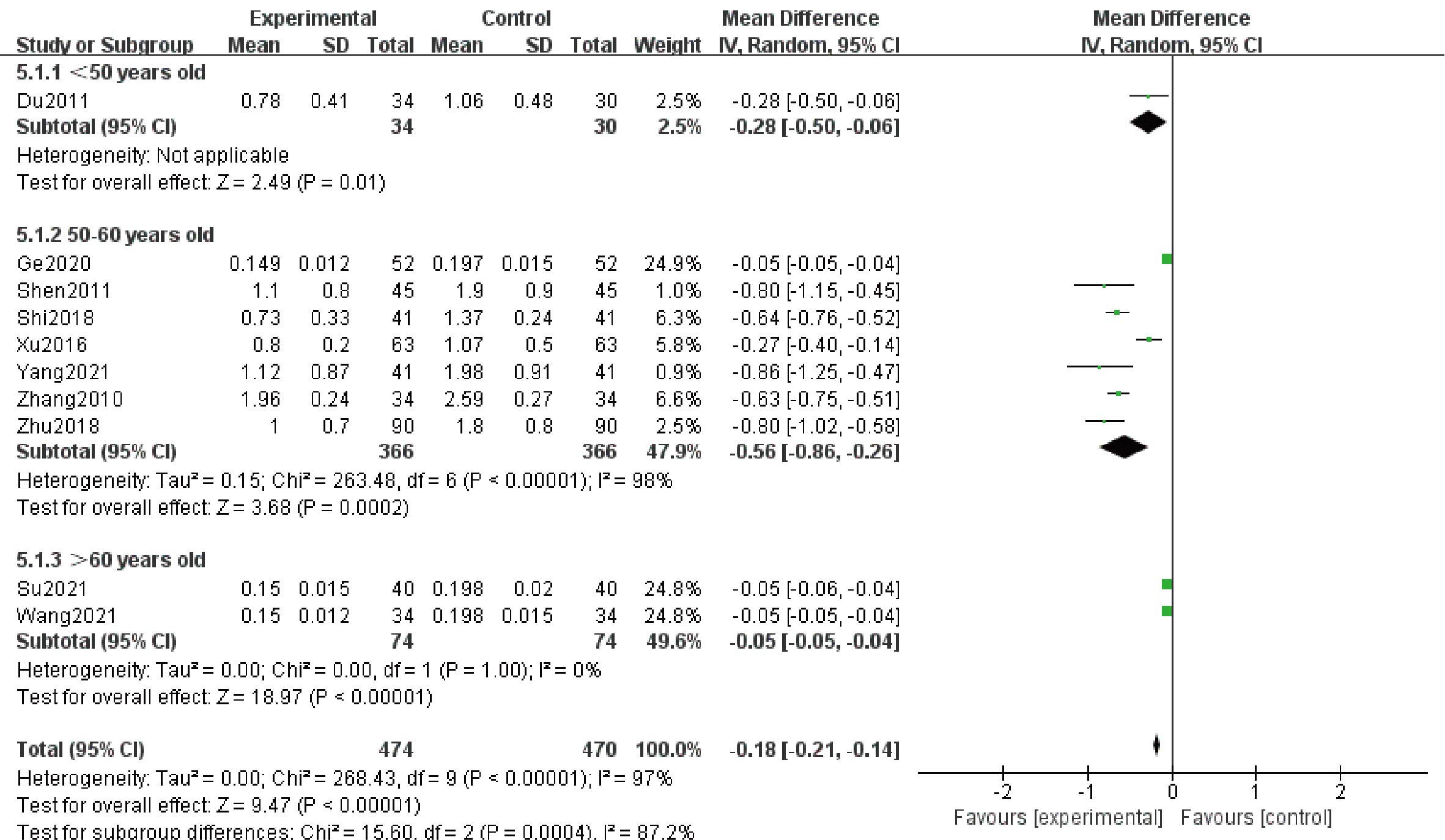

Supplement: Supplementary file 6 [file Image2.JPEG]

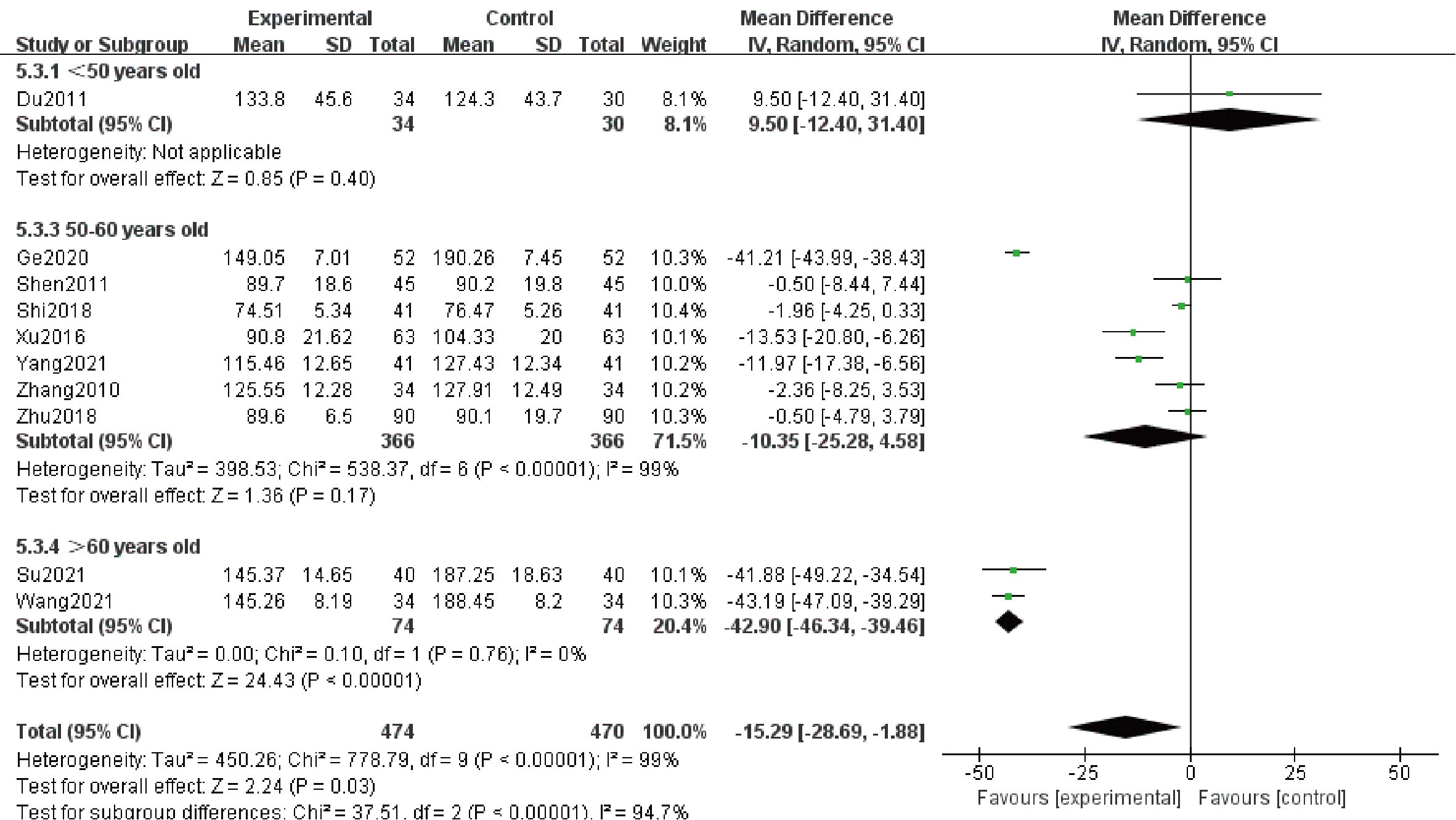

Supplement: Supplementary file 7 [file Image5.JPEG]

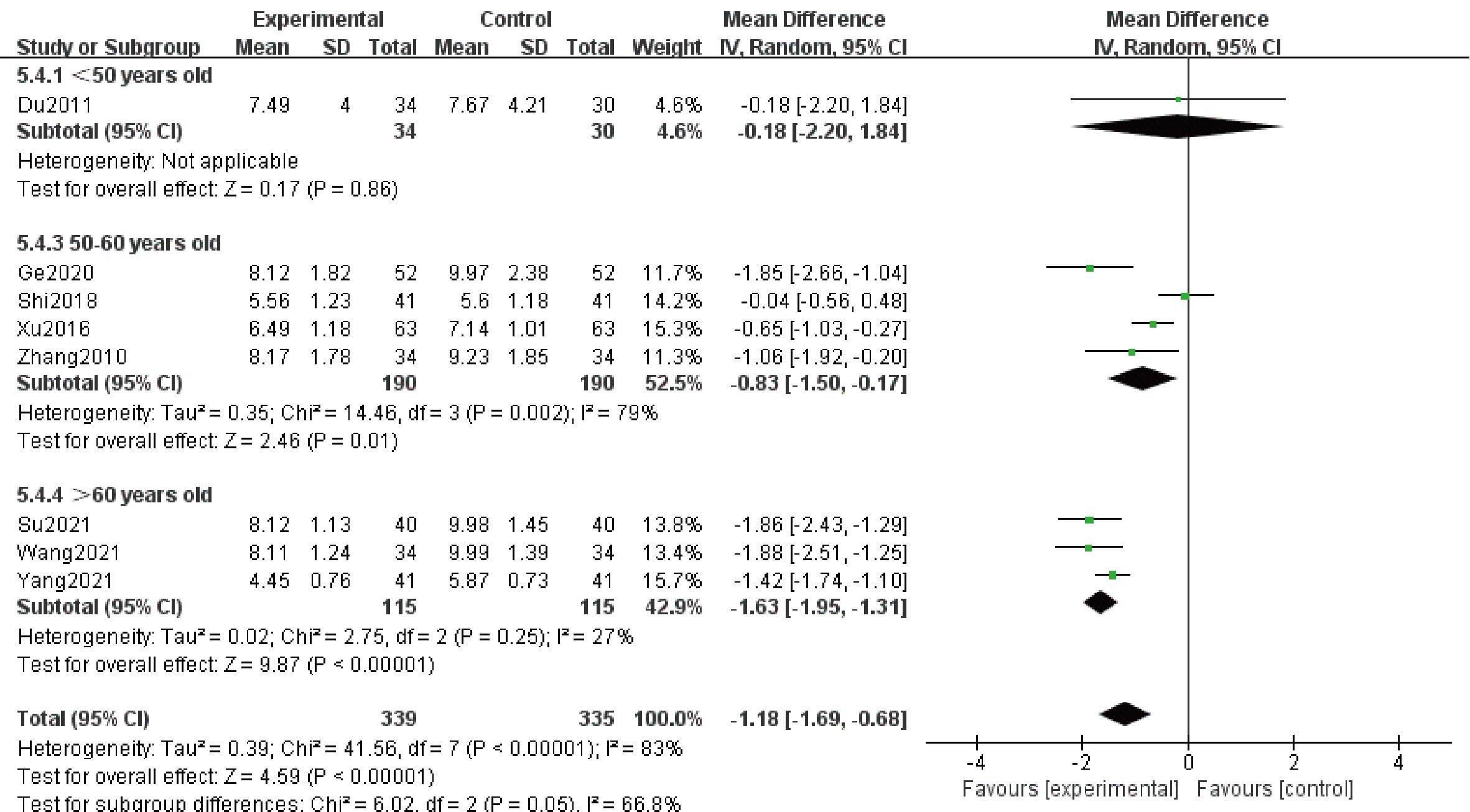

Supplement: Supplementary file 8 [file Image8.JPEG]

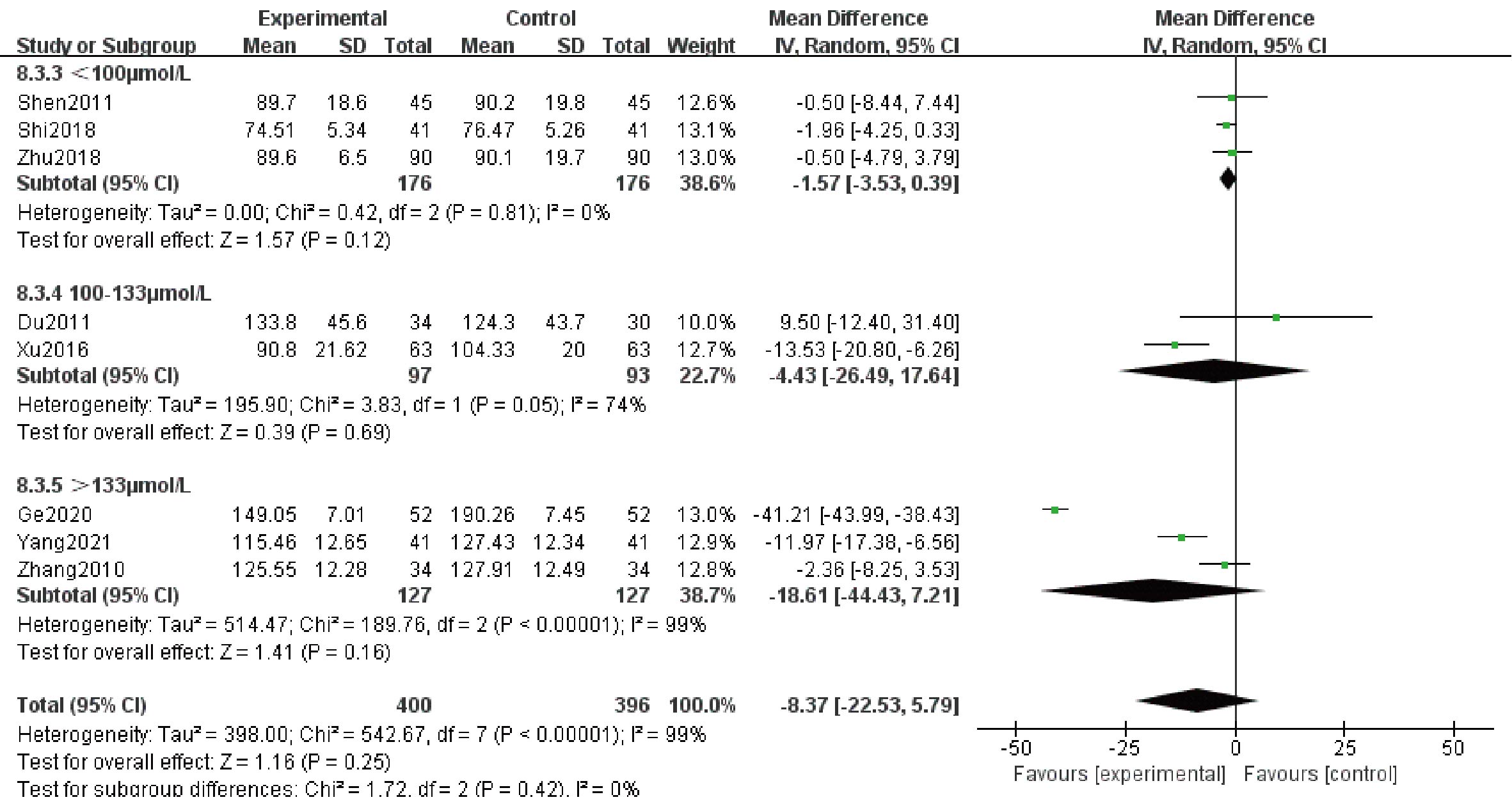

Supplement: Supplementary file 9 [file Image6.JPEG]
